# Supplementary material for: Filamentous fungus-produced human monoclonal antibody provides protection against SARS-CoV-2 in hamster and non-human primate models
Source: Nat Commun. 2024 Mar 14;15:2319. doi: 10.1038/s41467-024-46443-0 (PMC10940701; doi:10.1038/s41467-024-46443-0)

Supplementary material

A human monoclonal antibody produced in filamentous fungus provides protection from SARS-CoV-2 infection in a hamster and non-human primate model

Franziska K. Kaiser[1,11] Mariana Gonzalez Hernandez[1,11], Nadine Krüger[2,11], Ellinor Englund[3,11] Wenjuan Du[4], Anna Z. Mykytyn[5], Mathijs P. Raadsen[5], Mart M. Lamers[5], Francine Rodrigues Ianiski[6], Tatiana M. Shamorkina[6], Joost Snijder[6], Federico Armando[7], Georg Beythien[7], Malgorzata Ciurkiewicz[7], Tom Schreiner[7], Eva Gruber-Dujardin[2], Martina Bleyer[2], Olga Batura[2], Lena Erffmeier[2], Rabea Hinkel[2], Cheila Rocha[2], Monica Mirolo[1], Dubravka Drabek[8], Berend-Jan Bosch[4], Mark Emalfarb[9], Noelia Valbuena[9], Ronen Tchelet[9], Wolfgang Baumgärtner[7], Markku Saloheimo[3], Stefan Pöhlmann[2], Frank Grosveld[8], Bart L. Haagmans[5,11] & Albert D.M.E. Osterhaus[1,11]

[1]Research Center for Emerging Infections and Zoonosis, University of Veterinary Medicine, Foundation, Hannover, Germany; [2]German Primate Center - Leibniz Institute for Primate Research, Göttingen, Germany;[3]VTT Technical Research Centre of Finland Ltd., 02150 Espoo, Finland; [4]Virology Section, Infectious Diseases and Immunology Division, Department of Biomolecular Health Sciences, Faculty of Veterinary Medicine, Utrecht University, Utrecht, the Netherlands; [5]Department of Viroscience, Erasmus Medical Center, Rotterdam, the Netherlands; [6]Biomolecular Mass Spectrometry and Proteomics, Bijvoet Center for Biomolecular Research and Utrecht Institute of Pharmaceutical Sciences, Utrecht University, Padualaan 8, 3584, CH, Utrecht, The Netherlands; [7]Department of Pathology, University of Veterinary Medicine, Foundation, Hannover, Germany; [8]Department of Cell Biology, Erasmus Medical Center, Rotterdam, the Netherlands and Harbour BioMed, Rotterdam, the Netherlands. [9]Dyadic International, Inc., Jupiter, FL, USA; [10]Global Virus Network, Baltimore, MD 21201, USA.[11]These authors contributed equally: Franziska K. Kaiser, Mariana Gonzalez Hernandez, Nadine Krüger, Ellinor Englund, Bart L. Haagmans, Albert Osterhaus

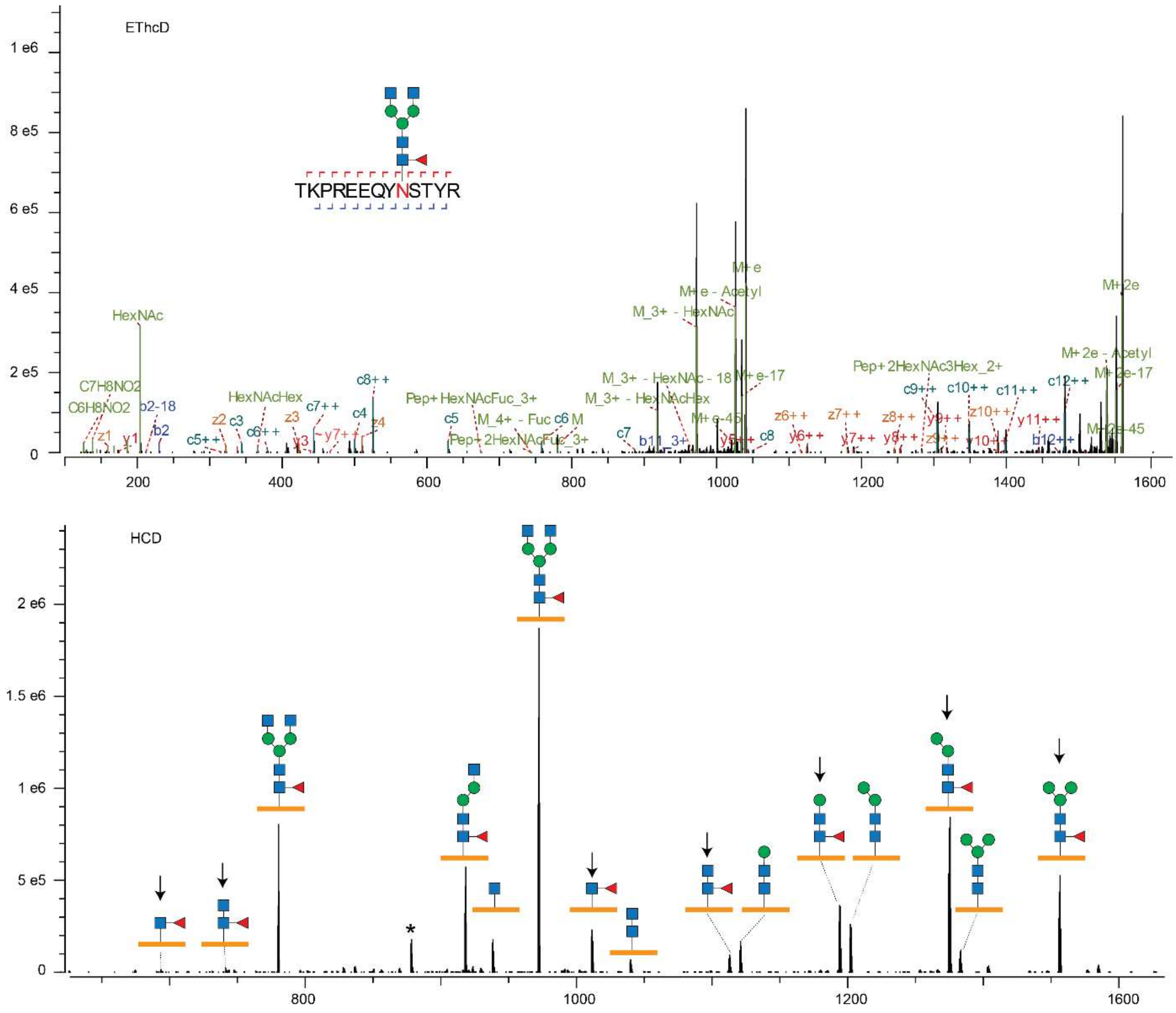

Supplementary Figure 1. Tandem mass spectrometry of fucosylated N-glycopeptide from HEK293 produced HuMab 87G7. Shown are an EThcD and HCD spectrum of the same glycopeptide precursor. The EThcD spectrum supports proper assignment of the intact glycan mass to the correct peptide sequence. The HCD spectrum contains many glycopeptide fragments supporting the presence of core fucosylation (arrows). There is one unassigned peak in the spectrum (asterisks), likely an internal fragment.

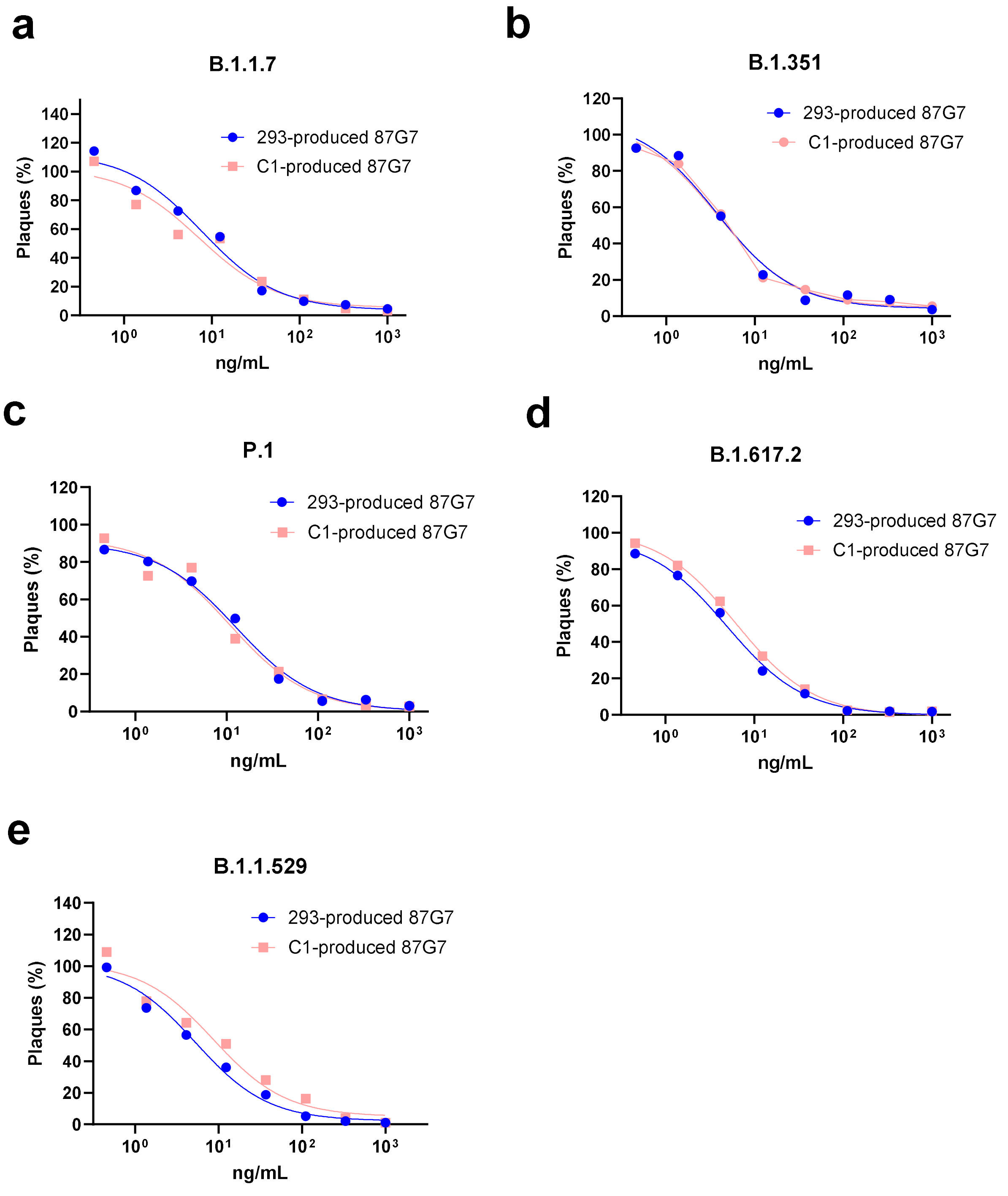

Supplementary Figure 2. C1 and HEK293T produced HuMab 87G7 bind and neutralize SARS-CoV-2 VOCS with comparable efficiency. Neutralizing activity of HuMab 87G7 against B.1..17. (a), B.1.351 (b), P1 (c), B.1.617.2 (d) and B.1.1.529 (e). The experiment was performed twice, data from a representative experiment are shown (*n* =2). Source data are provided as a Source Data file.

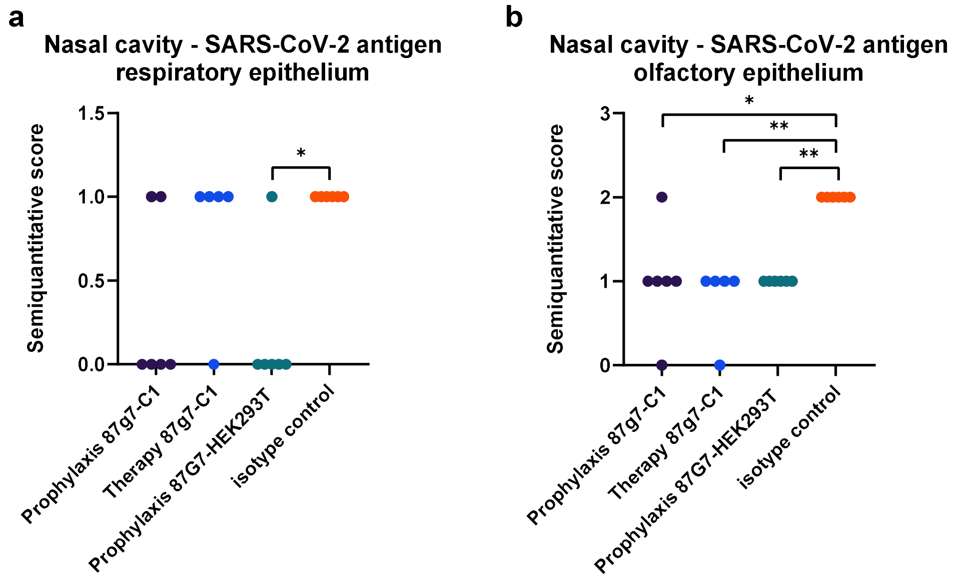

Supplementary Figure 3. IHC staining for SARS-CoV-2 nucleoprotein in nasal cavity from hamsters 4 days post infection. SARS-CoV-2 nucleoprotein was detected in (a) respiratory epithelium of the nasal cavity and (b) olfactory epithelium of the nasal cavity. *n* = 6 animals/group; */** indicate significant differences (P ≤ 0.05 and P ≤ 0.01 respectively); One-way ANOVA.

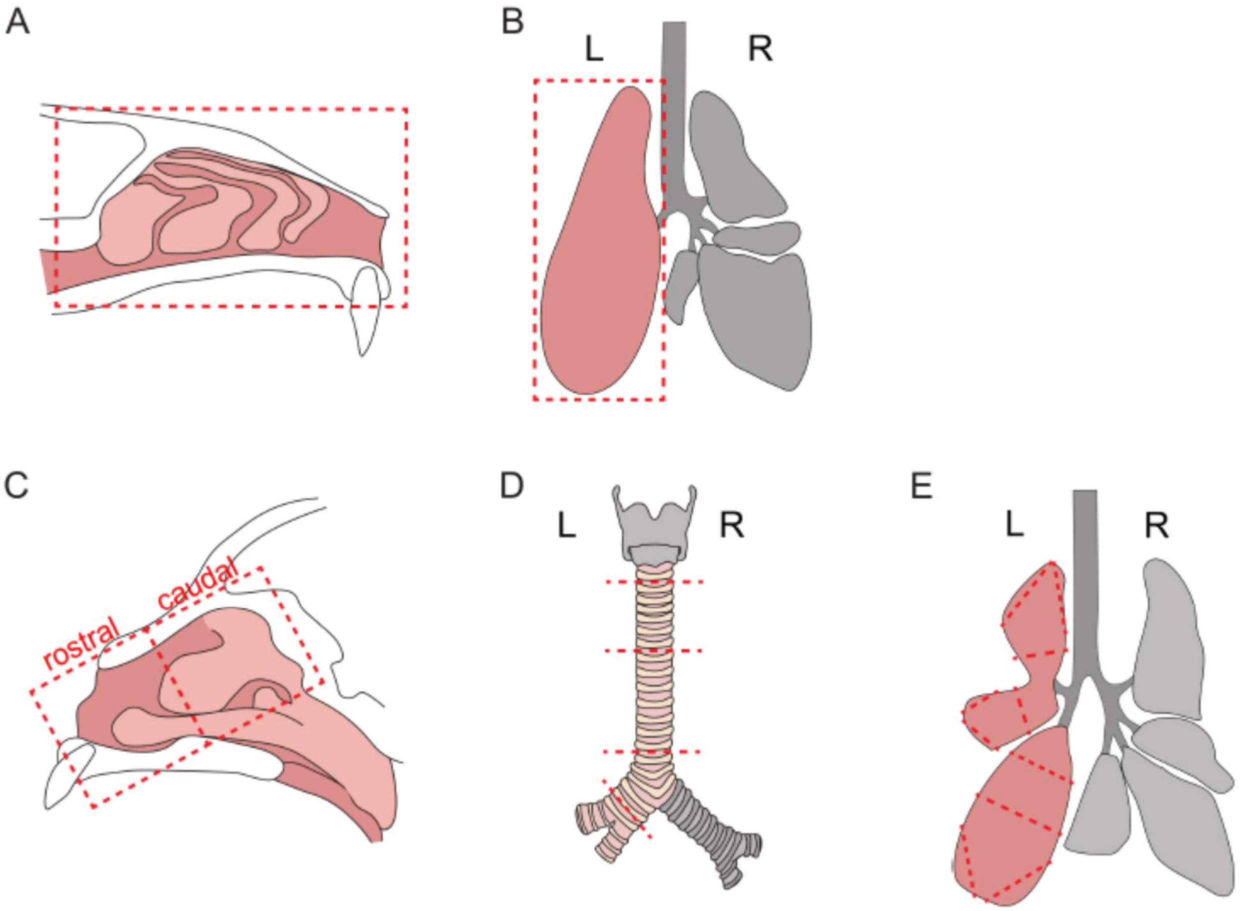

Supplementary Figure 4. Tissue trimming schematics for the evaluation of Syrian golden hamsters (A, B) and Rhesus macaques (C-E). The Evaluation of histopathological lesions and viral antigen expression was performed on total longitudinal sections of the left nasal cavity **(A)** and the entire left lung lobe **(B)** of Syrian golden hamsters. Evaluation of histopathological lesions and viral antigen expression in Rhesus macaques samples was performed on: **(C)** 1 slide containing the rostral portion of the nose, 1 slide containing the caudal part of the nose and 1 slide containing two cross sections of the nasal septum (not indicated in the panel); **(D)** 3 slides containing a cross section of the cranial, middle, and caudal trachea, 1 slide containing a cross section of the extra-pulmonary bronchus; **(E)** 10 areas of the lung were sectioned for analysis, 3 areas were divided in 2 slides for space reason, resulting in 13 slides in total for lung lesions evaluation.

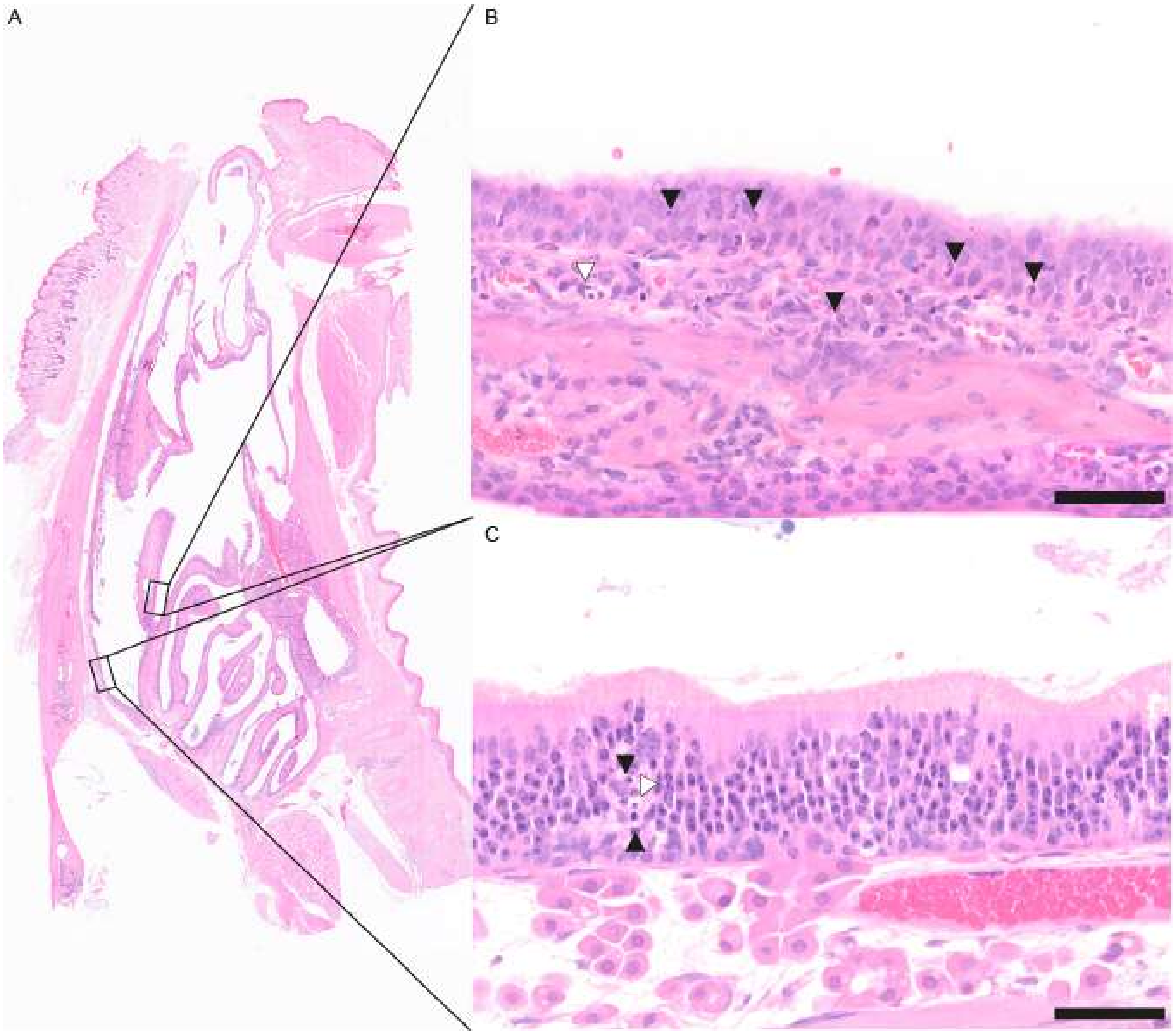

Supplementary Figure 5. Representative images of SARS-CoV-2 induced histopathological lesions in the nasal turbinates of Syrian golden hamsters (A-C). (A) Overview of the nasal cavity cross section. (**B)** Higher magnification of the respiratory epithelium displaying epithelial disorganization, cell death (white arrowhead), intraepithelial and subepithelial inflammatory infiltrates, characterized by macrophages and neutrophils (black arrowheads). **(C)** Higher magnification of the olfactory epithelium exhibiting epithelial disorganization, singe cell death (white arrowhead) and intraepithelial and inflammatory infiltrates, consisting of macrophages, lymphocytes and neutrophils (black arrowheads, (**C**)). Scale bars: 50 µm

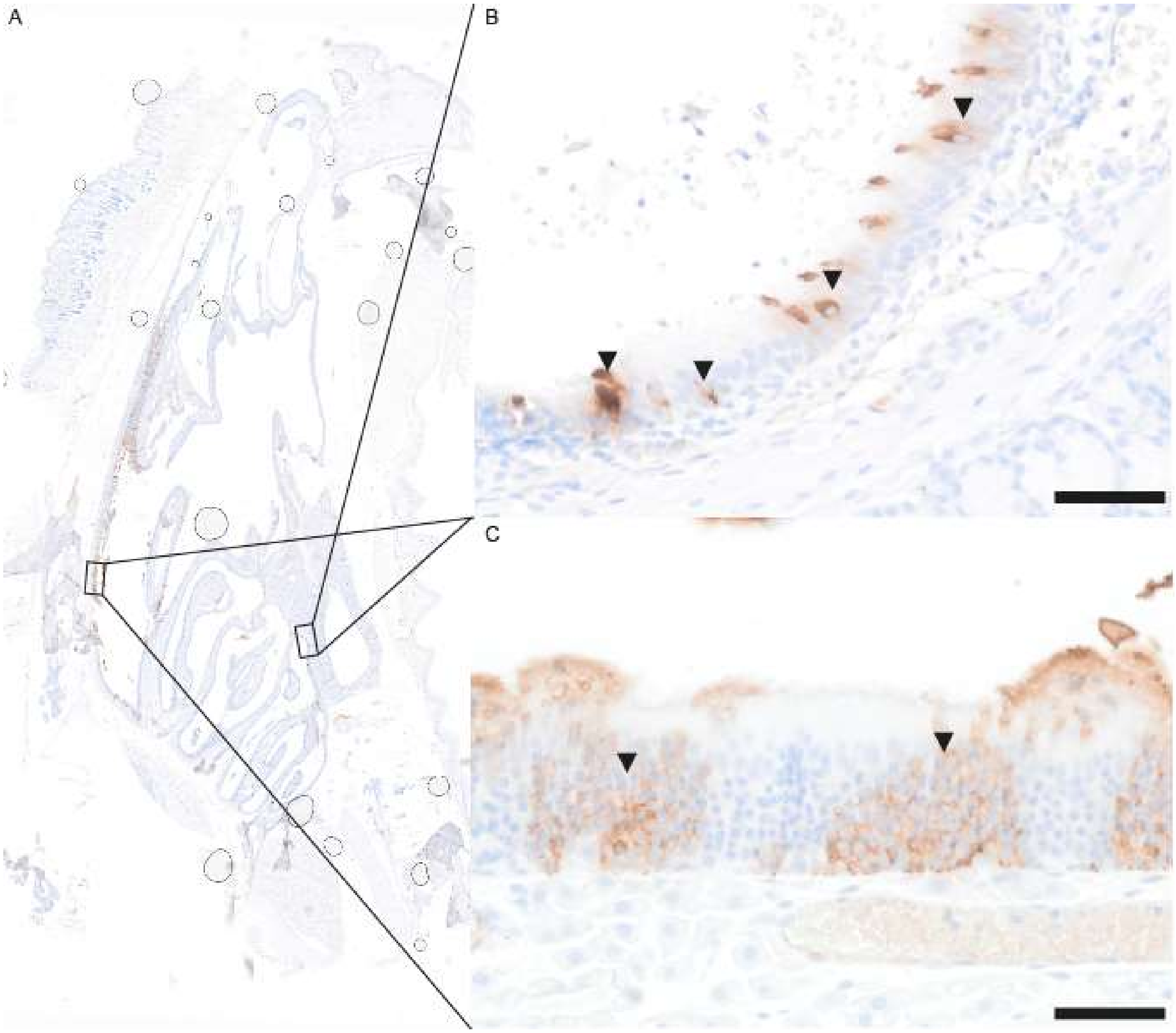

Supplementary Figure 6. Representative images of SARS-CoV antigen immunohistochemistry in nasal turbinates of of Syrian golden hamsters (A-C). (A) Overview of the nasal turbinates showing viral antigen (brown signal) in the epithelium and intraluminal exudate. **(B)** Higher magnification of the respiratory mucosa showing individual immunolabelled cells (arrowheads). **(C)** Higher magnification of olfactory mucosa showing viral antigen in many epithelial cells (arrowhead). Scale bars: 50 µm.

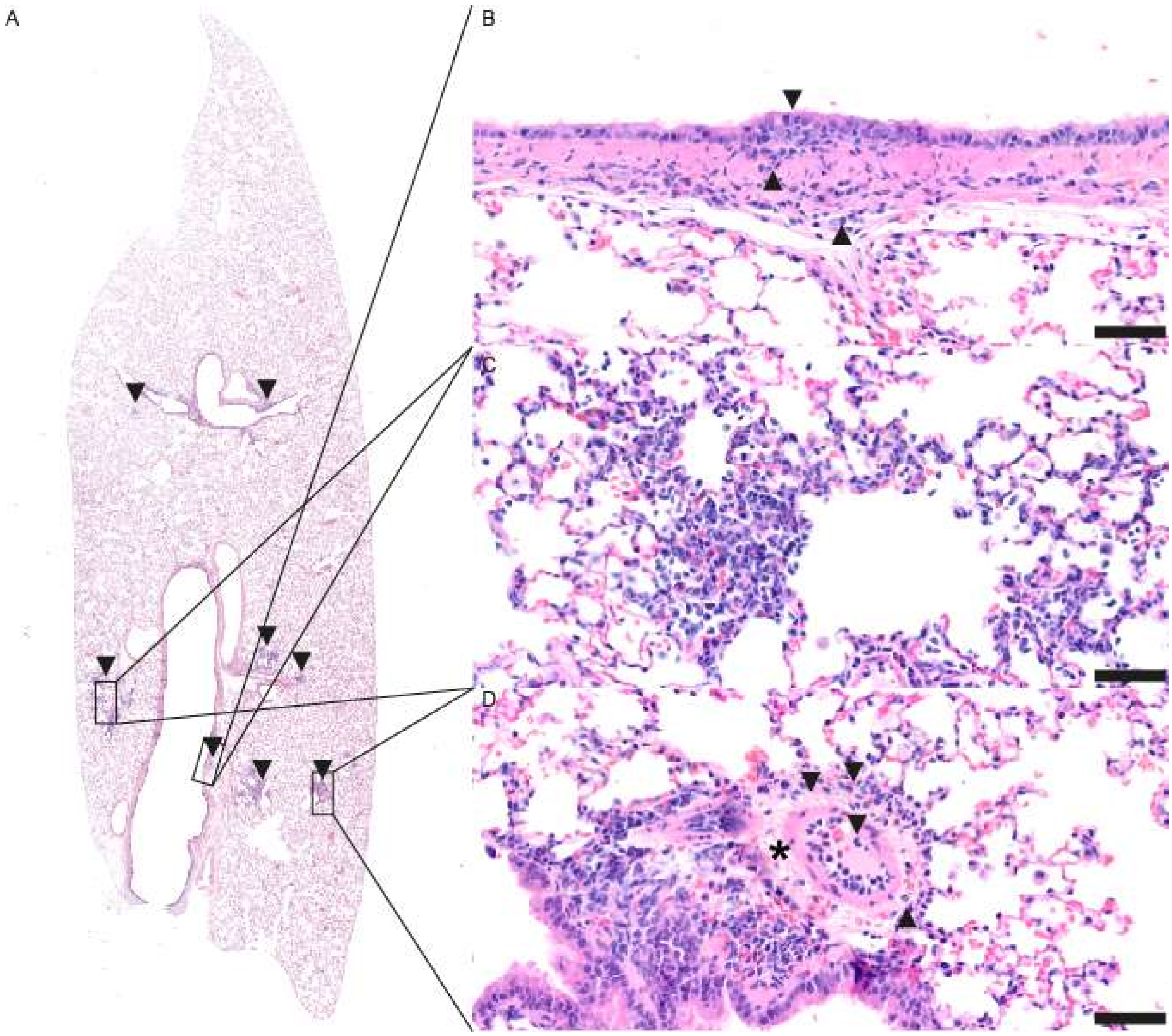

Supplementary Figure 7. Representative images of SARS-CoV-2 induced histopathological lesions in the lung of Syrian golden hamsters (A-D). A Overview of the left lung lobe showing multifocal consolidated areas (arrowheads). **B** Higher magnification of a bronchus with mild, focal intra- and subepithelial infiltrates (arrowheads) of lymphocytes and macrophages. **C** Higher magnification of alveoli displaying intraluminal and septal infiltrates mainly composed of macrophages and neutrophils. **D** Higher magnification of affected blood vessel with endothelialitis, mural and perivascular infiltrates (arrowheads) characterized by macrophages and neutrophils and perivascular edema (asterix). Scale bars: 50 µm.

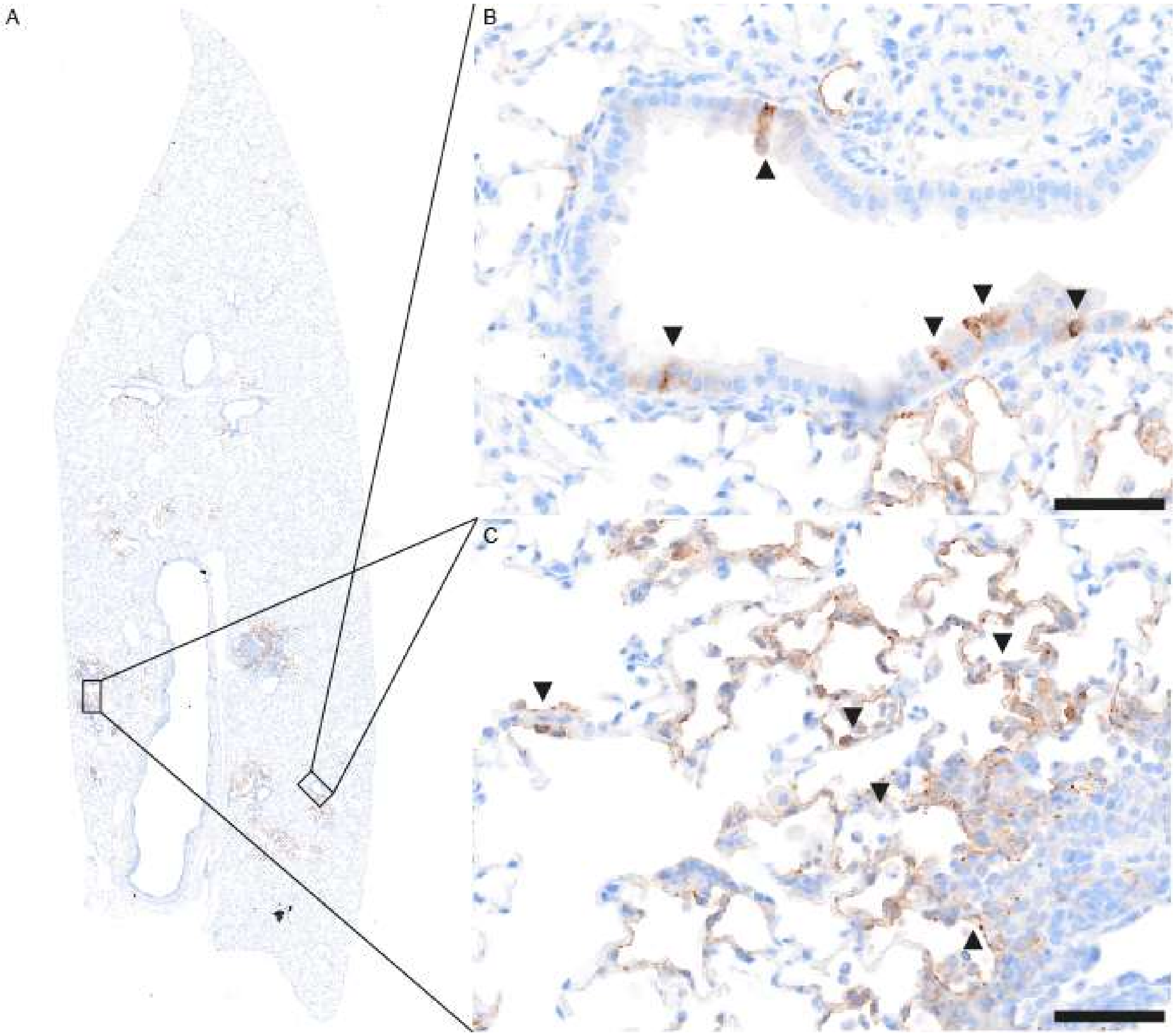

Supplementary Figure 8. Representative images of SARS-CoV antigen immunohistochemistry in lungs of hamsters (A-C). (A) Overview of the left lung lobe showing multifocal expression of viral antigen (brown signal). **(B)** Higher magnification of a bronchus with multiple individual immunolabelled cells (arrowheads). **(C)** Higher magnification of affected alveoli, showing numerous immunolabelled pneumocytes type 1 and single pneumocytes type II (arrowheads). Scale bars: 50 µm.

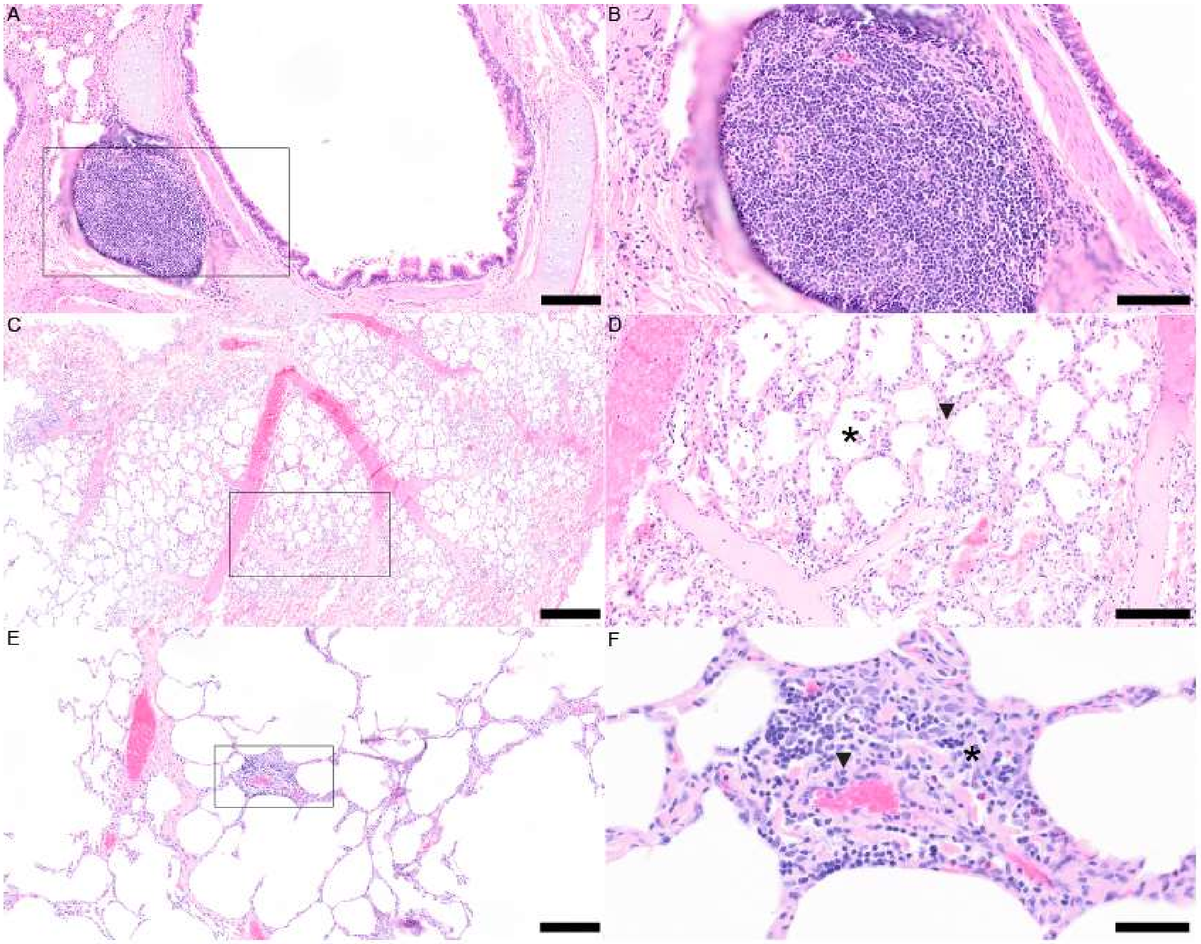

Supplementary Figure 9. Representative images of SARS-CoV-2 induced histopathological lesions in the lung of Rhesus Macaque (A) Overview of a main bronchus with associated lymphoid tissue (BALT) **(B)** Higher magnification of BALT showing a primary follicle structure composed of lymphocytes and histiocytes **(C)** Overview of a lung lobe showing multifocal consolidated areas (box). **(D)** Higher magnification of alveoli showing intraluminal (asterisk) and septal (arrowhead) infiltrates mainly composed of macrophages and neutrophils. **(E)** Overview of a lung lobe showing focal perivascular cuffing (box). **(F, box)** Higher magnification of vessel with endothelialitis (mitotic figure, arrowhead), mural and perivascular infiltrates composed of macrophages and neutrophils (asterisk). Scale bars A-C-E: 200 µm. Scale bars B-D: 50 µm. Scale bar F: 50 µm.

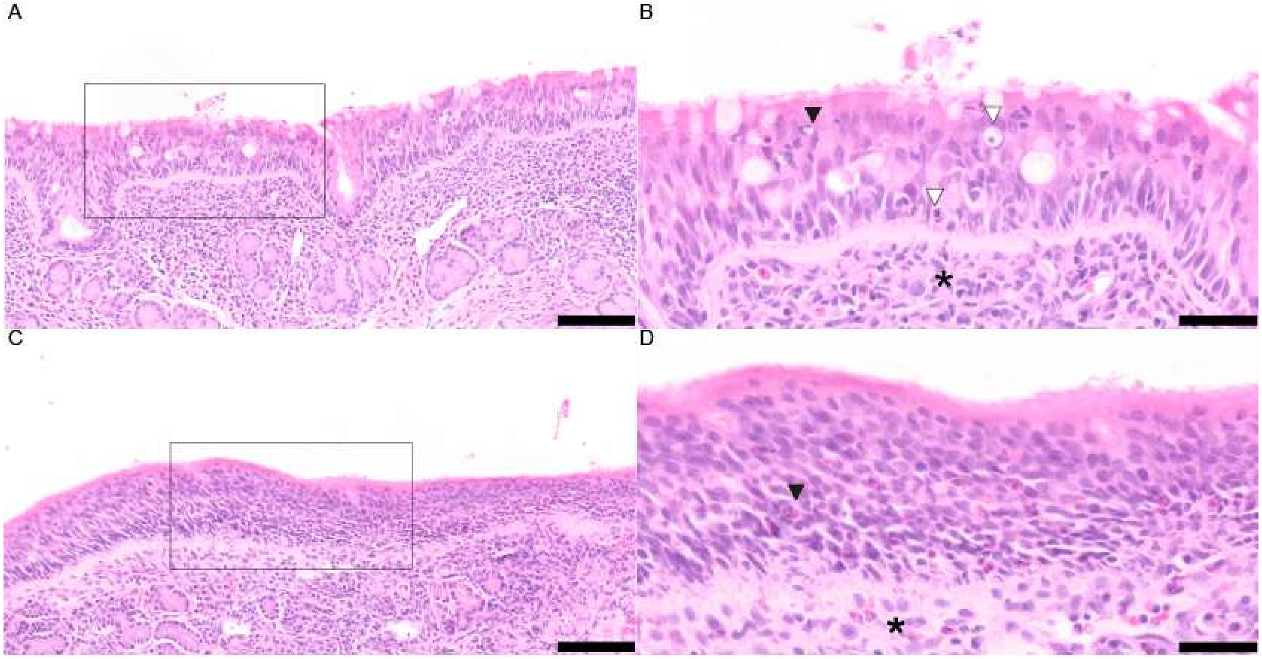

Supplementary Figure 10. Representative images of SARS-CoV-2 induced histopathological lesions in the nasal turbinates of Rhesus Macaque (A,C). B Higher magnification of the respiratory epithelium showing epithelial disorganization, cell death (white arrowheads), intraepithelial (black arrowhead) and subepithelial (asterisk) inflammatory infiltrates, composed of macrophages, lymphocytes and neutrophils. **D** Higher magnification of olfactory mucosa showing intraepithelial (black arrowhead) and subepithelial infiltrates of macrophages and neutrophils (asterisks) Scale bars A-C: 200 µm. Scale bars B-D: 50 µm.

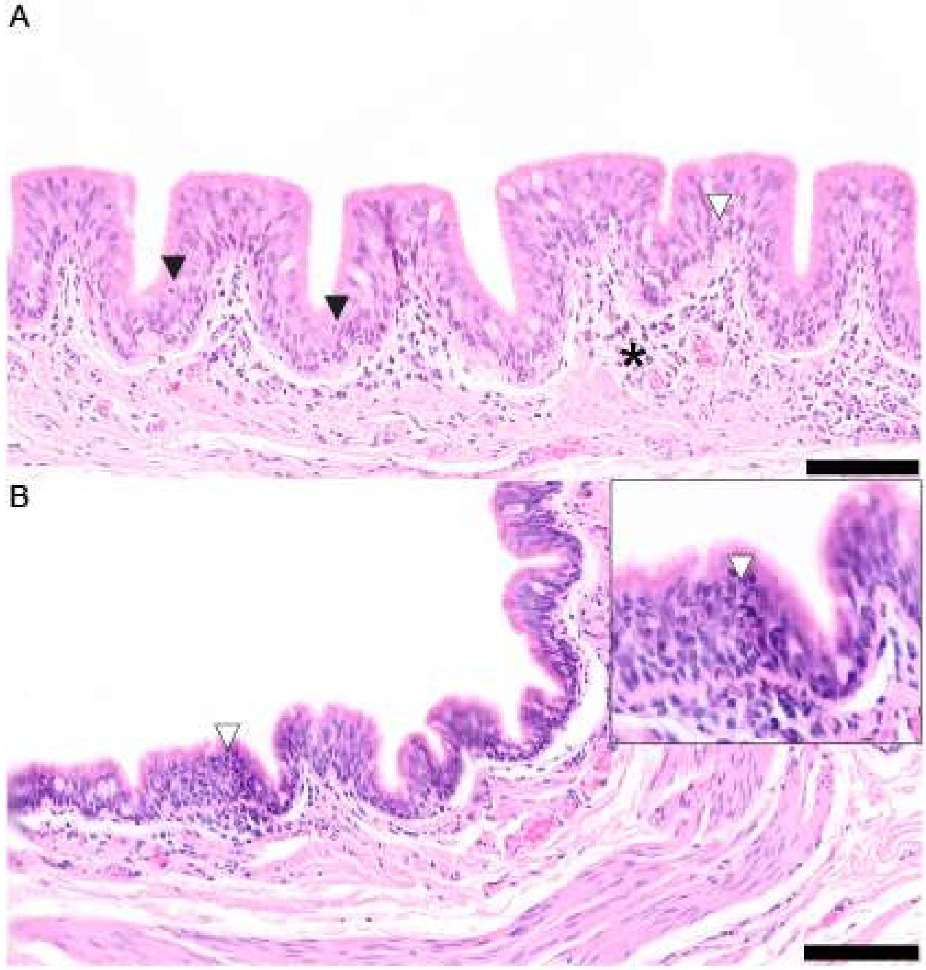

Supplementary Figure 11. Representative images of SARS-CoV-2 induced histopathological lesions in the extrapulmonary conductive airways of Rhesus Macaque (A) Tracheal respiratory epithelium showing cell death (black arrowheads), intraepithelial (white arrowhead) and subepithelial (asterisk) inflammatory infiltrates, composed of macrophages, lymphocytes and neutrophils. **(B)** extrapulmonary bronchial respiratory epithelium showing intraepithelial (white arrowhead) infiltrates of neutrophils (inset). Scale bars A-B: 200 µm.

Supplementary Table 1. List of oligonucleotide primers to check correct integration to the *cbh1* locus and the absence of the *cbh1* coding region.

Primer name	Sequence	Purpose
oMYT0157_cbh1_orf_for	GCTGACGCGAATGACACAG	forward primer for *cbh1* locus
oMYT0158_cbh1_orf_rev	CATGCCCTTGCCGTAGAAG	reverse primer for *cbh1* locus
oMYT0127_cbh1_5int_1	ATCAGACCACGACGGGAC	5 'end integration PCR forward
oMYT0087_bgl_pr_s4	GACTCTGAACGAGCCGGG	5' end integration PCR reverse
oMYT0130_cbh1_3int_2	CTCTAGTTAACGGCTACCTATTTAAGCT	3' end integration PCR reverse
oMYT0087_bgl_pr_s4	GACTCTGAACGAGCCGGG	5'end integration PCR forward

Supplementary Table 2. IC50 values of HEK293T and C1 produced HuMab 87G7 against different SARS-CoV-2 variants.

**IC50 (ng/ml)**	**614G**	**B.1.1.7**	**B.1.351**	**P.1**	**B.1.617.2**	**B.1.1.529**
293-produced 87G7	7.864	7.771	3.74	12.54	4.895	5.29
C1-produced 87G7	9.295	7.146	3.731	10.75	6.175	8.72

	Control group				Prophylactic group				Therapeutic high dose				Therapeutic low dose			
**Animal number**	DPZ_2878	DPZ_2866	DPZ_2978	DPZ_2921	DPZ_2995	DPZ_3037	DPZ_2955	DPZ_17199	DPZ_16954	DPZ_16997	DPZ_2956	DPZ_2985	DPZ_2754	DPZ_2768	DPZ_2918	DPZ_17155
Retropharyngeal lymph node	-	-	-	-	-	-	-	-	+	-	(+)	(+)	(+)	(+)	+	-
Pharyngeal mucosa	+	-	+	(+)	-	-	-	-	-	(+)	-	-	-	-	-	-
Trachea	(+)	+	-	-	-	-	-	-	-	-	(+)	(+)	+	-	-	-
Lung A1	(+)	-	-	(+)	-	-	-	-	-	-	-	-	-	-	+	-
Lung A2	-	-	-	(+)	-	-	-	-	(+)	-	(+)	(+)	-	-	+	-
Lung A3	(+)	-	-	-	-	-	-	-	-	-	(+)	-	-	-	-	-
Lung B1	-	-	-	-	-	-	-	-	-	-	(+)	-	-	-	+	-
Lung B2	-	-	-	-	-	-	-	-	-	-	(+)	-	-	-	(+)	-
Lung B3	-	-	-	-	-	-	-	-	-	(+)	+	-	-	-	+	-
Lung C1	-	-	-	(+)	-	-	-	-	(+)	(+)	-	-	-	-	-	-
Lung C2	-	-	-	-	-	-	-	-	(+)	-	(+)	-	(+)	+	-	+
Lung C3	-	-	-	-	-	-	-	-	-	-	(+)	-	(+)	-	+	-
Lung D1	-	-	-	(+)	-	-	-	-	-	-	-	-	-	-	-	-
Olfatory bulb	-	-	-	-	-	-	-	-	n.d.	n.d.	-	-	-	-	-	-
Tracheobronchial lymph node	-	(+)	-	-	-	-	-	-	-	+	-	-	(+)	(+)	+	(+)
Right main bronchus	(+)	-	-	-	-	-	-	-	(+)	(+)	-	-	-	(+)	-	-
Nasal turbinates (respiratory)	+	(+)	+	+	-	-	-	-	+	+	(+)	(+)	(+)	+	-	-
Nasal turbinates (olfactory)	(+)	-	+	+	-	-	-	-	(+)	(+)	-	(+)	-	+	-	(+)
Tonsil	-	+	-	(+)					-	-	-	-	-	-	-	-

Supplementary Table 3. Virus isolation from NHP 4 days post infection for different sample sites. Calu-3 cells were inoculated with tissue homogenates and five days later cells were fixed. Cytopathic effects of cells were confirmed by immunostaining against SARS-CoV-2. + isolation positive; (+) unclear result; - isolation negative

Figure 1. Uncropped

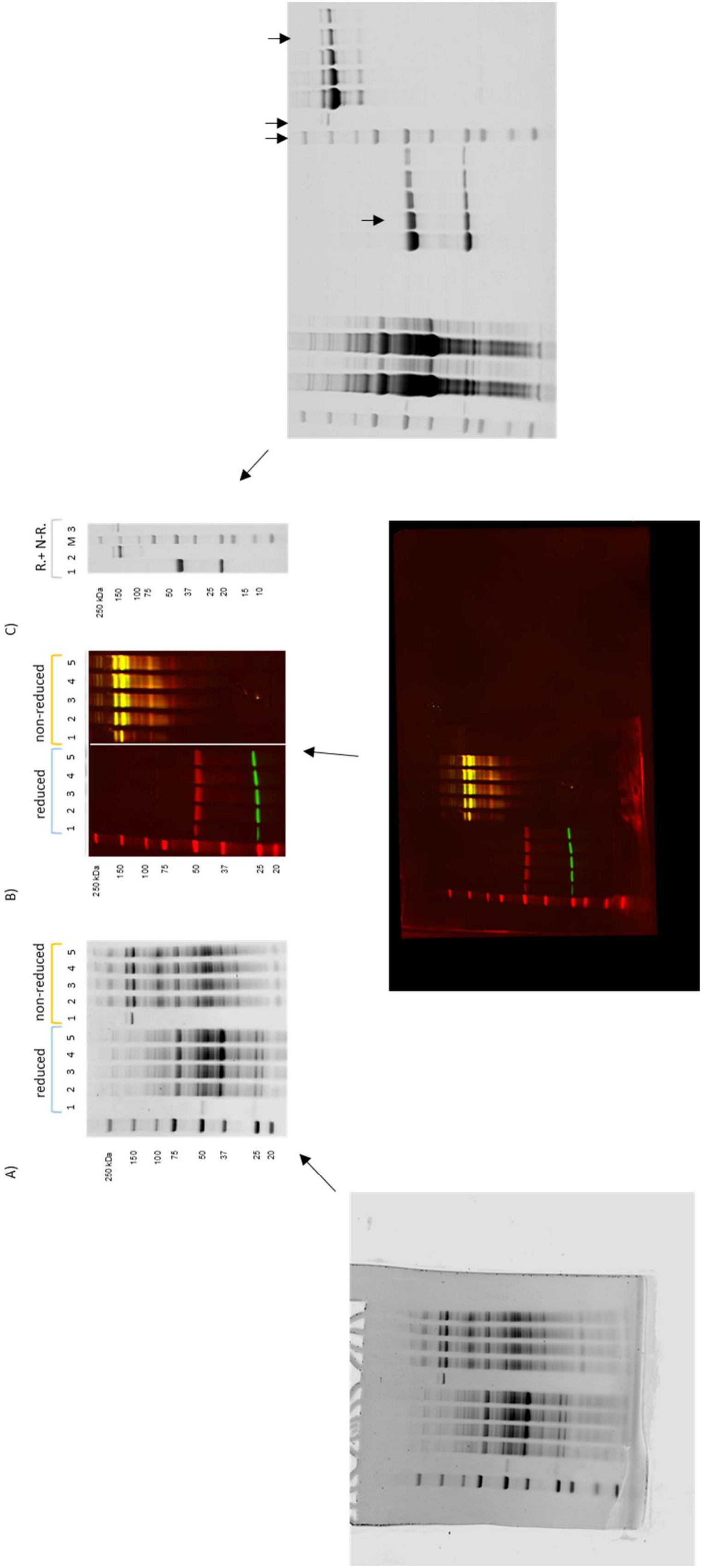

Supplement: Supplementary file 1 — Supplementary Information [file 41467_2024_46443_MOESM1_ESM.pdf]
